# Light Quality-Mediated Influence of Morphogenesis in Micropropagated Horticultural Crops: A Comprehensive Overview

**DOI:** 10.1155/2022/4615079

**Published:** 2022-12-02

**Authors:** Cunying Fan, Abinaya Manivannan, Hao Wei

**Affiliations:** ^1^Qufu Normal University, Qufu 273165, China; ^2^National Institute of Plant Genome Research, Aruna Asaf Ali Marg, New Delhi 110067, India

## Abstract

In plants, light quality plays significant roles in photomorphogenesis and photosynthesis. Efficient *in vitro* plant propagation techniques involve tailoring of various environmental cues and culture media according to the plant species. Plant tissue culture consists of several applications in scientific research, agriculture, biotechnology, and commercial industrial purposes. Utilization of light to enhance the quality of the *in vitro* raised plants have been evidenced by numerous researchers in plant tissue culture. The advent of light-emitting diode- (LED-) based artificial lighting systems in plant tissue culture for micropropagation has enhanced callus induction, shoot and root organogenesis, and acclimatization of *in vitro* propagated plants. Plants tend to perceive the light spectra present in the photosynthetically active region (PAR) ranging from 400 to 700 nm; this includes blue and red light wavelengths. Although the influence of spectral quality is being investigated in diverse plant species, particularly, its importance in *in vitro* propagated horticultural crops is gaining notable interest among researchers. In recent days, the application of LEDs provides better amenability according to the plant species of interest for efficient plant regeneration. Considering the growing necessity and emerging applications of LED supplemental lights for propagation of plants in *in vitro*, the present review summarizes the outcomes of various research studies dealing with LEDs in plant tissue culture. Moreover, the present endeavor has provided a comprehensive overview on the effects of LEDs in the morphogenesis of plants cultured *in vitro*.

## 1. Introduction

Tissue culture-based large-scale production of plants with selected traits has enhanced the cultivation of various plants with a wide range of economic values. It accelerates the production of genetically homogenous disease-free plants with favorable traits [1, 2]. Plant tissue culture serves as an indispensable tool for mass propagation of plants which are difficult to produce via conventional methods and also aids in the conservation of endangered plant species. Moreover, the micropropagation renders effective production of secondary metabolites in medicinal plants. Also, this technique acts as a vital tool for biotechnological applications such as cloning and transformation [2]. The plants propagated under an *in vitro* environment provide an opportunity to tailor the development of plants by influencing several diverse microenvironmental cues such as augmentation of plant growth regulators (PGRs), various chemical treatments, culture medium composition, and culture environments such as temperature, light, and humidity [3–5]. The tissue culture-based production of plants is economically valuable; for instance, the trade value of micropropagated seedlings was in the range of 50 billion USD which is increasing 15% per year [6]. In order to meet the international standards for trade and to produce high-quality *in vitro* propagules, various novel strategies have been devised by tailoring the plant tissue culture environment.

Light acts as the vital environmental cue that influences plant photosynthesis and photomorphogenesis. Light signals are involved in the regulation of physiology, growth, and other metabolic process in the following forms such as spectral quality, light intensity or quantity, and photoperiod or duration. Light spectral quality influences plants to unveil a high degree of physiological and biochemical malleability. Plants absorb light signals via photoreceptors such as cryptochrome, phytochrome, and phototrophins which influence the photomorphogenesis [7]. Among the light spectral qualities, plants significantly absorb the light spectrum that falls in the photosynthetically active region (PAR) ranging from 400 to 700 nm. The morphogenesis and physiology of plants are majorly affected by red and orange spectra (610-720 nm), by blue and purple in the range of 400-510 nm, and to some extent by yellow and green light in the spectral range of 510-610 nm [8]. Previous reports evidenced the light quality-mediated regulation of plant growth [9], secondary metabolite biosynthesis [10], and flowering [11]. Blue and red spectral ranges are widely researched due to their prominent involvement in the regulation of plant growth and development [12]. For instance, blue light receptors phototrophins have the ability to regulate the stomatal aperture movement and phototropism [13, 14]. Similarly, red light is effectively absorbed by the plant pigments such as chlorophyll and carotenoids which can influence the endogenous phytohormones and elicitation of secondary metabolites in plants [12].

Under *in vitro* plant growth conditions, the light is supplied by conventional lights such as fluorescent lamps and high-pressure sodium lamps. However, the broad range spectral distribution in these conventional lights results in inefficient availability of specific spectral qualities associated with photomorphogenesis [12]. Moreover, the conventional light sources require high electrical energy consumption and excess heat production [15]. Therefore, an efficient and amenable light source is necessary to improve the efficiency in plant tissue culture environment. Light-emitting diodes (LEDs) are considered as an effective substitute to traditional fluorescent lamps due to its versatile spectral quality, energy efficiency, narrow-spectrum illumination, less heat radiation, compactness, and longer life [16, 17]. Moreover, the optimization of spectral quality in an *in vitro* environment can positively influence the plant regeneration and growth. According to previous reports, the LEDs can be regulated dynamically, and it allows to determine the optimal composition and wavelengths of plant photoreceptors for the enhancement of *in vitro* grown plants [12, 18, 19]. Similarly, the LED spectra can influence the growth and development of plants by eliciting a cascade of physiochemical effects [20]. Numerous beneficial physiological modulations, such as improvement of photosynthesis [21], early flowering [22], secondary metabolite biosynthesis [23], and somatic embryogenesis [24], have been studied under LED application. Owing to the various advantages over conventional light sources, LEDs have gained a notable importance in plant tissue culture. Several reports have evidenced the benefits of LEDs in the different stages or aspects of *in vitro* propagation of horticultural crops as listed in Table 1.

**Figure 1:**
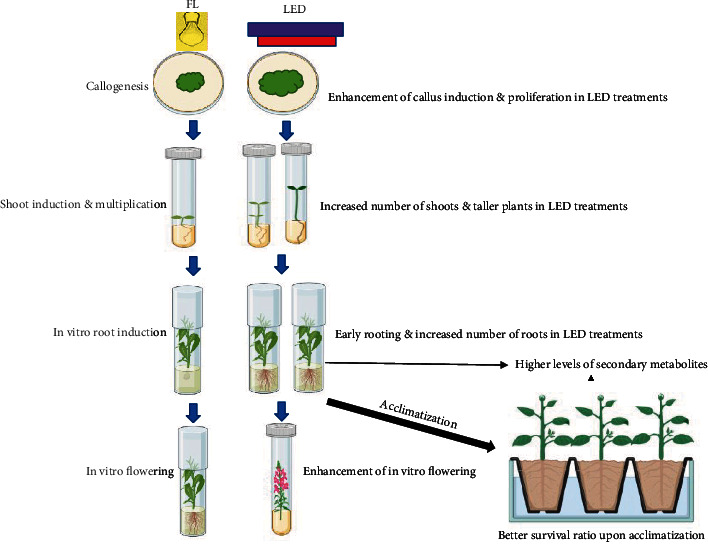
Schematic illustration of the application of LEDs in different stages of plant tissue culture.

Taken together, numerous reports are encouraging the utilization of LED lighting systems for plant growth. To perceive the global insight, the present review illustrates the application of LEDs in each step of plant tissue culture and its effect on morphogenesis (Figure 1).

## 2. Effect of LEDs on Callus Induction and Proliferation

*In vitro* plant regeneration consists of two primary initial stages of callusing including callus induction and proliferation. The callus induction is essential for indirect organogenesis and plant regeneration. Furthermore, callus induction plays a vital role in the investigation of reaction of plant cells to external factors in an *in vitro* environment and for the accumulation of specialized metabolites with pharmaceutical values [69]. Light quality strongly influences the onset of callus formation and proliferation [70]. Spectral quality-mediated regulation of callusing has been observed in different plants either under individual spectral quality or in combinations. For instance, the effects of red LED on callus induction and proliferation of *in vitro* plants have been observed in diverse horticultural crops [28, 70, 71]. Red light-mediated promotion of callus induction could be attributed by the increase in endogenous auxin levels in cotton [70]. Similarly, the application of red light also upregulated the expression of somatic embryo marker genes, increased activities of antioxidant enzymes, and higher accumulation of polyamines in in vitro cotton callus culture [70]. The regulation of endogenous hormones particularly auxins in the initial stage of callus formation by red light could be one of the molecular rationales behind the significant callus induction. Similarly, red light improved callus induction and differentiation in *Phalaenopsis* [26] and *Cymbidium* [27]. In Cymbidium, the red LED improved callus induction and formation from protocorm-like body (PLB) explants [27]. Similarly, the red LED treatment was effective for the maximum callus formation in *W. somnifera* [28]. According to Johkan true [29], red LED enhanced the biosynthesis and transportation of phytohormones in plants, which could have enhanced the induction of callus in *W. somnifera*. Stimulation of biomass in callus cultures of *Rhodiola imbricata* upon the illumination of red LEDs was reported by Kapoor true [30]. Furthermore, the blue LED-mediated enhancement of callus formation and proliferation was also evident in various horticultural plants such as *Cydonia oblonga* Mill [31], *Cistanche deserticola* [32], and anthurium (Budiarto [33]. Blue light-mediated enhancement of callus proliferation and development could be attributed by the influence of phytochromes and blue light-absorbing photoreceptors [27]. Besides red and blue LED, several studies also focused on the effects of other spectral lights on callus formation and growth. According to Soni and Swarnkar [36], the induction of callus and shoot buds was significantly enhanced by yellow spectra in *Vigna aconitifolia*. Similarly, Nhut true [37] also demonstrated that yellow light was beneficial for the proliferation of callus and higher biomass in *Panax vietnamensis*. Apart from the individual spectral qualities, the combination of two or more light qualities further improved the callus induction and proliferation in *in vitro* plant cultures [35]. Mixed red and blue LED was beneficial for callus proliferation. Recent report by Hassanpour [34] found that red-blue LED treatment was beneficial for callus production and secondary metabolism in *Hyoscyamus reticulatus*. Similarly, the maximum callogenic biomass and high concentrations of bioactive compounds in *Canavalia ensiformis* were reported in mixed red-blue light treatment [35]. Moreover, white LED enhanced the biomass of calli in *Solanum xanthocarpum* [38] and *Lepidium sativum* [39]. Hence, these results illustrate the influence of light quality in the initiation and proliferation of callus in *in vitro* plants. Moreover, the differential outcomes of the spectral regime could be due to the varied cellular physiological state and its interaction with the environmental cues such as light [33]. However, further elucidation of precise mechanism behind callus induction and proliferation by different light quality needs to be unraveled.

## 3. Effects of LEDs on the Induction and Growth of Shoots

*In vitro* plant regeneration via shoot organogenesis is an important step in the micropropagation process. Establishment of optimal shoot induction and multiplication involves several factors such as the composition of culture medium with optimal concentrations of cytokinin and auxins, regeneration efficiency of the explant, and culture environment. The application of different spectral qualities functions diversely on the formation and growth of shoots in *in vitro* culture. The ideal wavelengths of LEDs to plant photoreceptors can enhance the induction, proliferation, and differentiation of shoots [20]. The effects of LEDs vary in species and developmental stage-dependent manner, and the molecular mechanism behind the differential response is unclear [22]. Blue LED displayed positive effects in shoot induction and growth in micropropagated horticultural crops. In *Paphiopedilum delenatii* the blue LED treatment increased the regeneration rate of shoots [46]. In a similar manner, the blue light increased the number of shoots regenerated in *Cattleya intermedia* × *C. aurantiaca* [47], *Ajuga multiflora* [44], *Anthurium andreanum* [33], *Curculigo orchioides* [48], *Rosa kordesii* [49], and *Brassica napus* [50]. The light quality-mediated effects in shoot induction vary among the species and cultivars. The variation in genetic composition, differential expression of photoreceptors upon different light quality treatments, endogenous hormone levels, and other physiological factors of the plant species could contribute to the different photomorphogenic responses [12, 22]. For example, the micropropagated carnation “Green Beauty” produced taller plants under red LED, whereas the red LED treatment did not produce significant effects on shoot elongation in “Purple Beauty” cultivar of carnations [52]. According to Yorio true [51], the blue LED treatment significantly increased the shoot length with higher number of nodes in lettuce. The blue light has been reported to enhance the endogenous cytokinin levels [72]. Cytokinins are vital hormones pivotal for shoot induction and regeneration under in vitro conditions. Therefore, application of blue light could have triggered the endogenous cytokinin levels which improved the shoot regeneration in various plant species. On the contrary, the repressive effects of red LED on shoot induction were also illustrated in some plants. The red LED treatment displayed the least shoot organogenesis percentage and number of shoot buds in *C. orchioides* [48]. However, the inhibitory effects of red light in shoot induction of some plants remain elusive [48]. In *Scrophularia takesimensis* Nakai, red LED treatment produced taller plants in comparison with blue LED and fluorescent light treatments [40]. Similar effects of red LEDs were reported in the micropropagated Chrysanthemum [41], *Rehmannia glutinosa* [42], grape [43], *Ajuga multiflora* Bunge [44], and *Oncidium* [45]. The application of red light reduced the shoot induction capacity in some plants but significantly increased the nodal length and stem elongation. Some studies have reported the effect of red light for the discharge of apical meristem and development of shoot primordia which could have facilitated the elongation of stem [73].

Therefore, the incorporation of mixed LED wavelengths can be considered as a promising option in the propagation of plants in an *in vitro* environment. The *Anthurium andreanum* plants produced a higher number of adventitious shoot regeneration under mixed blue and red LEDs [53]. Similarly, in *Vanilla planifolia* the blue and red light combination in 1 : 1 ratio significantly enhanced the shoot numbers per explant and biomass [54]. Likewise, the application of red and blue LEDs in 1 : 2 ratio promoted shoot growth in *Dendrobium officinale* [55]. The application of 90% red LED and 10% blue LED encapsulated strawberry shoots developed optimally under *in vitro* conditions [56]. Furthermore, the red and blue combination increased the growth and activity of peroxidase enzyme in *in vitro* shoot cultures of *Phoenix dactylifera* [57]. Apart from the red and blue, other spectral qualities like yellow LED influenced the shooting. Supplementation of yellow light resulted in increased shoot proliferation, higher number of shoots per explant, the longest shoot length, and the highest biomass of shoots in orchids Billmore et al. [58]. The positive effects of yellow light can be due to the occurrence of some photoreceptors and the overlap switch between the spectral regions such as green, orange, and red that could be channeled to modulate the physiology and morphogenesis of plants [74]. However, in *Plectranthus scutellarioides*, the green light produced lower shoot dry mass in comparison with other light treatments [64]. Contrarily, green LED at the highest PPFD increased shoot elongation in lettuce [29]. Thus, based on the above-mentioned findings, the monochromatic LEDs and their combinations have a profound influence on shoot regeneration. In addition, light quality functions variously in species-specific or cultivar-dependent manner [22].

## 4. Effects of LEDs on the Induction and Growth of Roots

To achieve successful acclimatization and a high survival rate, rooting of plants *in vitro* is of immense importance. The significance of light quality is also vital for the formation of roots during *in vitro* propagation. The improvement of rooting under LED was discovered in *Tripterospermum japonicum* [75], highbush blueberry microcuttings [76], and *Jatropha curcas* L. [77]. Moreover, the root activity of upland cotton grown under red LED light displayed the highest root activity than other treatments [59]. Likewise, red LED enhances rooting in several horticultural crops such as *Ficus benjamina* [78], grape [43], anthurium [33], and *Morinda citrifolia* [60]. Red light significantly influences endogenous auxin hormones which could have affected the rooting positively in the *in vitro* plants. In similar manner, blue LED-mediated improvement of rooting has been evidenced in various plants. For instance, in *Vitis vinifera*, blue light improved the rooting percentage [61]. Blue LED significantly increased root length in lettuce [51], augmented adventitious rooting in cherry rootstock [62], improved the root induction in wheat [63], induced root growth, and promoted the root length and number of roots in *Rehmannia glutinosa* [42]. In contrast, blue LED inhibited rooting in birch, *Prunus serotina*, and *Tripterospermum japonicum* [75, 79, 80]. Blue light-mediated rooting response can be due to the combinatorial effect of blue light-absorbing photoreceptors and related genes [62, 81]. Recently, the application of blue light in combination with auxin NAA improved the adventitious rooting by influencing the rooting-related genes [81]. Particularly, blue light increased the expression of LBD transcription factors which regulates the adventitious rooting in plants [81].

In addition, combinations of different spectral mixtures on rooting of plants *in vitro* were also investigated by several researchers. For instance, [50]) reported that the shoots of *Brassica napus* cultured under blue-red LED mixture in 3 : 1 ration displayed longer roots which correlated with the maximum survival ratio. In *Oncidium*, the combination of red-blue-far red spectral qualities enhanced the rooting and biomass in comparison with the monochromatic red, blue, and far red [45]. According to Budiarto [33], mixture of red-blue spectral qualities particularly with higher red light proportion significantly augmented rooting in Anthurium, whereas, in *Plectranthus scutellarioides*, the combination of red-green LED treatment increased rooting and biomass in comparison to the white light treatment [64]. Moreover, in *Cunninghamia lanceolata*, total combination of red-blue-purple-green in 72.1 : 9.1 : 9.1 : 9.1 ratio boosted the rooting in comparison with other treatments [8]. The impact of light quality on rooting varies for different species. Thus, it is necessary to select appropriate monochromatic LEDs or mixed LEDs used in the rooting stage of *in vitro* culture to enhance the root induction in tissue culture-raised plants.

## 5. Effects of LEDs on the Growth of Plantlets during Acclimatization

The final stage of *in vitro* culture is marked by successful survival of *in vitro* regenerated plants with well-grown roots. In this process, the well-rooted *in vitro* plants will be transplanted and exposed to the natural environment. Nevertheless, the acclimatization of micropropagated plants is affected by diverse *in vitro* environmental characteristics such as high relative humidity, less intensity of light compared to field conditions, ingredients of growing media with sucrose, and other phytohormones [82]. Even though various factors exist, light plays a key regulator which affects morphogenesis and photosynthesis which directly influence the growth and development of tissue culture-derived plants [83]. According to Woźny and Miler [84], the application of LEDs improved acclimatization of microshoots to ex vitro conditions. For instance, the combination of red and blue LEDs in the ratio of 80 : 20 significantly enriched the survival and acclimatization of *Spathiphyllum* in comparison to fluorescent light (FL) [65]. Likewise, the positive effects of LEDs during acclimatization have been observed under ex vitro conditions. Several reports evidenced the utilization of LEDs as the light source during acclimatization, which benefitted the rate of survival of plantlets raised *in vitro*. Similarly, the micropropagated *Coffea canephora* grown under LEDs acclimatized well in comparison with plants grown under conventional FL [85]. In a similar manner, the supplementation of LEDs enhanced the acclimatization response in grapevine [86]. In strawberry, LED treatments significantly enhanced the acclimatization, fresh and dry weight, and vegetative growth [56]. According to Ferreira et al. [87], the sugarcane cultures grown under LED illumination augmented the survival ratio. The sugarcane cultures maintained in LED treatments displayed higher antioxidant enzyme activities in comparison with FL which aided in the ROS balance during the initial stages of acclimatization [87]. Hence, the significant improvement of survival ratio of plants grown under LED treatments can be due to the enhancement of rooting and mediation of antioxidant enzymes by LED application in *in vitro* plantlets. These findings suggest that the application of LED light improves both *in vitro* growth and development which in turn increases the acclimatization to natural environment.

## 6. Effects of LEDs on *In Vitro* Flowering

Generally, flowering is an intricate mechanism triggered by various genetic, biochemical, and environmental factors. Investigation of *in vitro* flowering can offer an ideal platform to study flower induction and development, which can be utilized in breeding programs [88, 89]. Light quality is vital factor that affects flowering. Several reports have evidenced the significant effects of light quality in different plant species. However, very few studies have utilized LED-mediated investigation of *in vitro* flowering. The cryptochrome-mediated induction of early flowering upon blue LED treatment in *Arabidopsis thaliana* was reported by Eskins [66] and Lin [67]. However, according to Victorio and Lage [68], the maximum rate of flowering was observed under white light treatment in *Phyllanthus tenellus.* On the other hand, the blue LED enhanced the frequency of *in vitro* flowering in S*crophularia takesimensis*, a potential medicinal plant, but the red LEDs significantly reduced the flowering frequency in comparison with fluorescent light [40]. Moreover, the red LED-mediated inhibition of *in vitro* flowering was also illustrated in *Phyllanthus tenellus* [68] and *Euphorbia milii* [90]. Based on the above investigations in different plants, flowering is highly influenced by photoreceptors particularly phytochromes and cryptochromes. Blue and red lights could induce or suppressed the flowering genes via their receptors which in turn accelerated or inhibited *in vitro* flowering in different plant species depending upon the genetic and physiological factors [91]. The above findings delineate the vital role of LEDs in the flowering process. Further, these reports also demonstrate the possibility of conducting LED irradiation system to improve the flowering in plants, particularly ornamental plants, regenerated *in vitro*.

## 7. *In Vitro* Photomorphogenic Variations upon Different Light Quality Treatments

Light is a vital photon source which is converted into chemical energy by plants perceived by the photoreceptors. Supplementation of light energy by LEDs in *in vitro* environment can facilitate the morphogenesis by providing uniform spectral distribution which can be optimally absorbed by the photoreceptors. For instance, in several plants, the application of blue and red LEDs separately or in combinations enhanced the photosynthesis competence and improved the assimilation of carbon dioxide via photosynthesis [92, 93]. However, the molecular rationale behind the photomorphogenesis plasticity exhibited by plants upon different LED treatments is scarcely studied. Spectral quality influenced the expression of genes associated with biosynthesis of amino acids, nucleic acids, and fatty acids which are potential for plant regeneration and development under *in vitro* environment [94–96]. Light quality can regulate the endogenous hormones in the micropropagated plants by regulating structural and regulatory genes involved in various metabolic pathways, physiological process such as photosynthesis, cell wall biosynthesis, and secondary metabolism necessary for the plant regeneration and development in *in vitro* conditions [12]. According to Li true [96], *in vitro* regenerated grape plants' specific spectral qualities increased explicit gene expressions. In detail, the blue light enhanced the expression of genes associated with the synthesis of microtubules, pigment biosynthesis, sugar metabolism, and genes associated with resistance [96], whereas red light greatly influenced the expression of genes related to defense response in grapes [96]. In *in vitro* grown *Arabidopsis thaliana*, the gene expression analysis upon different spectral quality treatments illustrated that the different light treatments regulated the differential expression of cell wall-related genes such as expansin, xyloglucan endotransglycosylase, and glucanases [97].

Previous studies evidenced the impact of light quality in each stage of *in vitro* plant propagation as discussed in the above sections. The supplementation of red or blue light treatments significantly influenced the induction or proliferation of callus. Possible molecular mechanism behind the callus induction by red lights can be due to the interaction of phytochromes, activities of antioxidant enzymes, polyamines, and higher accumulation of endogenous auxins which plays vital role in the initiation of callus [70]. Similarly, the improvement of shoot induction by blue light spectra can be attributed by enhancement of endogenous cytokinin observed in *in vitro* plants [72]. Likewise, *in vitro* rooting upon light quality treatments benefitted the survival ratio in various micropropagated plants. The regulation of endogenous hormones and expressions of genes associated with rooting might be the primary mechanism behind the LED-mediated augmentation of *in vitro* rooting. Further, regulation of antioxidant metabolism during the acclimatization stage also contributed to the better survival of plants grown under LEDs. In addition, the difference in the photomorphogenesis of *in vitro* plants by different LEDs can be attributed to photoreceptor-based transcriptional regulation of potential genes and enzymes involved in vital pathways associated with endogenous hormone signaling, cell wall biosynthesis, photosynthesis, and primary and secondary metabolism. However, in-depth research on vital functional genes associated with the light quality-based plant morphogenesis needs to be unveiled in the future using next-generation sequencing and biotechnological approaches.

## 8. Conclusions

The utilization of light quality for *in vitro* propagation as a cue to enhance the physiology and bioactive compounds has attracted considerable research interest among plant biologists, and it can improve the commercial micropropagation systems for horticultural crops in a large scale. As discussed in the above sections, majority of the positive effects of LEDs are attributed to the compatibility of the wavelengths perceived by photoreceptors in the plants. The morphological and growth enhancement of *in vitro* propagated plants by the application of specific or mixed LED treatments will result in higher efficacy in comparison with the conventional light sources used in an *in vitro* environment. Moreover, the flexibility of wavelengths in LED-based systems can be exploited to provide appropriate wavelengths in a plant- or species-specific manner in micropropagation systems to produce high-quality plant materials.

## Figures and Tables

**Table 1:** Effects of different light qualities on physiology and morphogenesis of micropropagated horticultural crops.

Serial number	Species name	Light quality	Improved trait	Reference
1	*Scrophularia kakudensis*	Red	Enhancement of secondary metabolites	[25]
2	*Phalaenopsis*	Red	Callus induction	[26]
3	*Cymbidium*	Red	Callus induction	[27]
4	*Withania somnifera*	Red	Callus formation	[28]
5	*Withania somnifera*	Red	Callus induction	[29]
6	*Rhodiola imbricata*	Red	Biomass improvement	[30]
8	*Cydonia oblonga* Mill	Blue	Callus formation and proliferation	[31]
9	*Cistanche deserticola*	Blue	Callus formation and proliferation	[32]
10	*Anthurium andreanum*	Blue	Callus formation and proliferation	[33]
11	*Hyoscyamus reticulatus*	Mixed red and blue	Callus production and secondary metabolism	[34]
12	*Canavalia ensiformis*	Mixed red and blue	Enhancement of callogenic biomass and elicitation of bioactive compounds	[35]
13	*Vigna aconitifolia*	Yellow	Enhancement of callus induction and shoot buds	[36]
14	*Panax vietnamensis*	Yellow	Callus proliferation and higher biomass	[37]
15	*Solanum xanthocarpum*	White	Enhancement of callus biomass	[38]
16	*Lepidium sativum*	White	Enhancement of callus biomass	[39]
17	*Scrophularia takesimensis*	Red	Taller plants	[40]
18	Chrysanthemum	Red	Taller plants	[41]
19	*Rehmannia glutinosa*	Red	Taller plants	[42]
20	*Vitis vinifera*	Red	Taller plants	[43]
21	*Ajuga multiflora* Bunge	Red	Taller plants	[44]
22	*Oncidium*	Red	Taller plants	[45]
23	*Paphiopedilum delenatii*	Blue	Shoot regeneration	[46]
24	*Cattleya intermedia* × *C. aurantiaca*	Blue	Increased number of shoots	[47]
25	*Ajuga multiflora*	Blue	Increased number of shoots	[44]
26	*Anthurium andreanum*	Blue	Increased number of shoots	[33]
27	*Curculigo orchioides*	Blue	Increased number of shoots	[48]
28	*Rosa kordesii*	Blue	Increased number of shoots	[49]
29	*Brassica napus*	Blue	Increased number of shoots	[50]
30	*Lactuca sativa*	Blue	Increased in shoot length with higher number of nodes	[51]
31	*Dianthus caryophyllus*	Red	Taller plants	[52]
32	*Anthurium andreanum*	Mixed blue and red	Adventitious shoot regeneration	[53]
33	*Vanilla planifolia*	Mixed blue and red	Enhancement of shoot numbers per explant and biomass	[54]
34	*Dendrobium officinale*	Mixed blue and red	Shoot growth	[55]
35	*Fragaria x ananassa*	Mixed blue and red	Optimal development of encapsulated strawberry shoots under in vitro conditions	[56]
36	*Phoenix dactylifera*	Mixed red and blue	Increased growth and activity of peroxidase enzyme in in vitro shoot cultures	[57]
37	Orchids	Yellow	Increased shoot proliferation	[58]
38	*Lactuca sativa*	Green	Increase in shoot elongation	[29]
39	Upland cotton	Red	Enhancement of in vitro rooting	[59]
40	*Ficus benjamina*	Red	Enhancement of in vitro rooting	[94]
41	*Vitis vinifera*	Red	Enhancement of in vitro rooting	[43]
42	*Anthurium andreanum*	Red	Enhancement of in vitro rooting	[33]
43	*Morinda citrifolia*	Red	Enhancement of in vitro rooting	[60]
44	*Vitis vinifera*	Blue	Enhancement of in vitro rooting	[61]
45	*Lactuca sativa*	Blue	Increased root length	[51]
46	Cherry	Blue	Increased adventitious rooting	[62]
47	Wheat	Blue	Improvement in root induction	[63]
48	*Rehmannia glutinosa*	Blue	Induced root growth and promoted the root length	[42]
49	*Brassica napus*	Mixed blue and red	Longer roots and maximum survival ratio	[50]
50	*Oncidium*	Combination of red-blue-far red	Enhancement of the rooting and biomass	[45]
51	*Anthurium andreanum*	Mixed red and blue	Augmented rooting	[33]
52	*Plectranthus scutellarioides*	Mixed red and green	Increase in rooting and biomass	[64]
53	*Cunninghamia lanceolata*	Combination of red-blue-purple-green	Increased rooting	[8]
54	*Spathiphyllum*	Combination of red and blue	Increase in survival and acclimatization	[65]
55	*Arabidopsis thaliana*	Blue	Early flowering	[66, 67]
56	*Phyllanthus tenellus*	White	In vitro flowering	[68]
57	S*crophularia takesimensis*	Blue	Enhancement of in vitro flowering	[40]
58	*Phyllanthus tenellus*	Red	Inhibition of in vitro flowering	[68]
59	*Euphorbia milii*	Red	Inhibition of in vitro flowering	[95]

## Data Availability

No data were used to support this study.
